# Measurement of extracellular volume fraction by cardiac magnetic resonance imaging detects diffuse myocardial fibrosis in systemic sclerosis

**DOI:** 10.1186/1532-429X-14-S1-O28

**Published:** 2012-02-01

**Authors:** Daniel C Lee, Roberto Sarnari, Alejandro Aquino, Maria M Izquierdo-Gomez, Brandon Benefield, Monique Hinchcliff, John Varga, Sofia Podlusky, Maria Carr, Aya Kino, James Carr, Edwin Wu, Sanjiv Shah

**Affiliations:** 1 Division of Cardiology, Department of Medicine, Northwestern University, Feinberg School of Medicine, Chicago, IL, USA; 2 Feinberg Cardiovascular Research Institute, Northwestern University, Feinberg School of Medicine, Chicago, IL, USA; 3 Division of Cardiology, Department of Medicine, Washington University Medical School, St. Louis, MO, USA; 4 Division of Cardiology, Department of Medicine, Hospital Universitario de Canarias, Tenerife, Spain; 5 Division of Rheumatology, Department of Medicine, Northwestern University, Feinberg School of Medicine, Chicago, IL, USA; 6 Department of Radiology, Northwestern University, Feinberg School of Medicine, Chicago, IL, USA

## Summary

We measured extracellular volume fraction (Ve) from pre- and post-contrast T1 maps of the left ventricle in 13 patients with systemic sclerosis (SSc) and 13 age-matched controls. SSc patients and controls were similar with regard to LV and RV mass, volumes, and function. However, Ve was significantly higher in SSc patients than in controls, even when patients with visible late gadolinium enhancement were excluded. Ve correlated with SSc severity as measured by the modified Rodnan Skin Score. Ve may be valuable for detection of myocardial involvement in SSc, even when conventional CMR appears normal.

## Background

Primary cardiac involvement is common in systemic sclerosis (SSc) and responsible for 25% of deaths. Myocardial extracellular volume fraction (Ve), derived from cardiac magnetic resonance (CMR) T1 mapping of the myocardium, has been shown to quantify diffuse myocardial fibrosis (DMF) - but its utility in SSc has not been studied. We hypothesized that subjects with SSc have a higher Ve compared to controls and that patients with worse SSc severity have higher Ve.

## Methods

CMR was performed in 13 SSc patients (5 diffuse and 8 limited cutaneous) and 13 age-matched controls. Cine, pre- and post- contrast T1 mapping, and late gadolinium enhanced (LGE) imaging was performed. LV mass index (LVMi), LV end-diastolic volume index (LVEDVi), LV ejection fraction (EF), RV mass index (RVMi), RV end-diastolic volume index (RVEDVi), RV ejection fraction (RVEF) and LGE as a percent of the LV (LGE%) were quantified (Medis QMass MR 7.2). Ve was calculated as Ve = [ΔR1myocardium/ΔR1bloodpool × p × (1 - hematocrit)] - Vp, where R1 = 1/ T1, ΔR1 is post-contrast - precontrast R1, p is myocardial specific density (1.05), and Vp is myocardial plasma volume fraction (0.045). Skin involvement was quantified in all SSc patients using the Modified Rodnan Skin Score (mRSS) by clinicians blinded to all CMR data.

## Results

LGE was visible in 3/13 SSc and 0/13 controls. Ve was significantly higher in SSc than controls, even when patients with visible LGE were excluded (Table 1A). In contrast, there was no significant difference between SSc and controls with regards to LVEF, LVMi, LVEDVi, RVMi, RV EDVi, or RVEF (Table 1B). Ve correlated significantly with mRSS in SSc patients (figure).

**Table 1:** Quantitative CMR in SSC and controls

Table 1A			
		Ve% (mean ± SD)	Compared to Controls

All SSc Patients (n = 13)		27.4 ± 4.6	p = 0.0003
SSc without LGE (n = 10)		26.9 ± 4.0	p = 0.001
Controls (n = 13)		20.6 ± 3.3	NA

Table 1B			

	SSc (n = 13) (mean ± SD)	Control (n = 13) (mean ± SD)	

LV Mass Index (g/m^2^)	39.8 ± 8.4	42.8 ± 5.9	p = 0.3
LV EDV Index (ml/m^2^)	69.4 ± 17.1	76.1 ± 16.2	p = 0.3
LV EF (%)	59.9 ± 9.2	57.0 ± 5.1	p = 0.3
RV Mass Index (g/m^2^)	29.6 ± 10.0	25.9 ± 10.5	p = 0.4
RV EDV index (ml/m^2^)	75.2 ± 25.7	73.4 ± 20.8	p = 0.9
RV EF (%)	47.8 ± 15.2	52.8 ± 7.8	p = 0.3
LGE (% of LV)	2.6 ± 8.0	0.0 ± 0.0	p = 0.3

**Figure 1:**
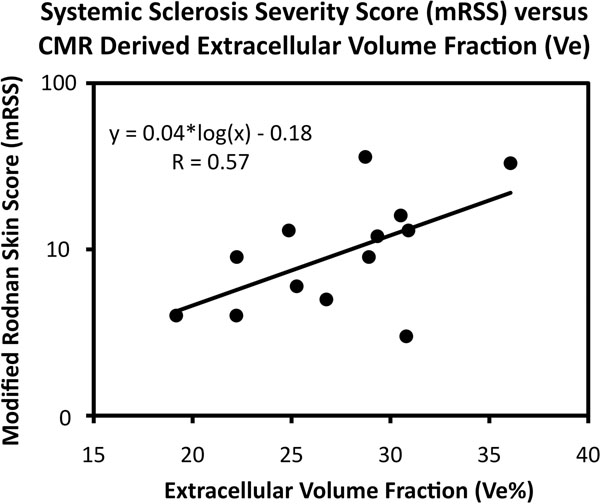


## Conclusions

This is the first study to demonstrate the utility of CMR T1 mapping for identification of diffuse myocardial fibrosis in SSc. Extracellular volume fraction measured by CMR correlates with SSc severity measured by mRSS. Ve identifies diffuse myocardial fibrosis in SSc patients, even in the absence of LGE. Given the high mortality associated with clinically symptomatic myocardial involvement in SSc, this technique may be valuable for detection even when conventional CMR appears normal.

## Funding

None.

